# Accuracy of Dengue, Chikungunya, and Zika diagnoses by primary healthcare physicians in Tegucigalpa, Honduras

**DOI:** 10.1186/s12879-023-08346-1

**Published:** 2023-06-01

**Authors:** María Fernanda Ávila Mejía, Pei-Yun Shu, Dar-Der Ji

**Affiliations:** 1 International Health Program, National Yang Ming Chiao Tung University, Taipei, Taiwan, R.O.C.; 2 Center for Diagnostics and Vaccine Development, Taiwan Centers for Disease Control, Ministry of Health and Welfare, Taipei, Taiwan R.O.C.; 3 Department of Tropical Medicine, National Yang Ming Chiao Tung University, Taipei, Taiwan, R.O.C.

**Keywords:** Dengue, Chikungunya, Zika, Clinical diagnosis, DBS, Accuracy

## Abstract

**Background:**

Dengue, Chikungunya, and Zika are co-endemic in Honduras and are often misdiagnosed due to similar clinical and epidemiological behavior. Most arboviral infections reported in primary care are based on clinical diagnoses without laboratory confirmation. Therefore, the accuracy of physicians’ diagnoses and the factors that affect them needs to be evaluated.

**Methods:**

A cross-sectional study with convenience sampling at primary healthcare centers was conducted from June to September 2016 and 2017. Clinical data and dried blood spots on Whatman 903 filter paper from 415 arboviral cases and 248 non-arboviral febrile cases were collected. Viral RNA was extracted from a 6-mm DBS paper disc and confirmed by RT-qPCR and sequencing.

**Results:**

Only 30.84% of diagnostic accuracy was observed in physicians in primary care when comparing arboviral clinical diagnosis with RT-qPCR detection. Moreover, in Dengue and Zika clinical cases, only 8.23% and 27.08% were RT-qPCR confirmed, respectively. No Chikungunya cases were confirmed. In 2017, 20.96% of febrile cases were RT-qPCR confirmed arboviral infections. The symptoms of 45.5% of arboviral cases can fit more than one case definition for arboviruses. The “symptom compliance” and “patient with suspected close contact” were the criteria most utilized by physicians for arboviral diagnosis. The pattern of the epidemiological curves of the arboviral clinical cases didn’t match the one of the RT-qPCR confirmed cases.

**Conclusions:**

Low diagnostic accuracy for overall and individual arboviral infections was observed in physicians. Unspecific symptomatology, overlapping case definitions, and reported close contact to an arboviral patient might contribute to misdiagnosis. Without laboratory confirmation, surveillance data may not reflect the real behavior of these diseases and could impact health interventions.

**Supplementary Information:**

The online version contains supplementary material available at 10.1186/s12879-023-08346-1.

## Background

Dengue, Chikungunya, and Zika are global concerns due to their increasing incidence, accelerated geographical expansion, and co-circulation [1]. Dengue is the most common mosquito-borne disease worldwide, with an estimated 50–100 million cases annually. Dengue’s first epidemic in Honduras occurred in 1978, with outbreaks occurring every 2 to 5 years [2]. Its high morbidity and mortality overwhelm the Honduran health system [3]. Chikungunya was an emerging disease that rapidly expanded into the American Continent in 2013 [4] and was identified in Honduras in 2014. During a 2015 Chikungunya outbreak, 235 children were hospitalized with neurological complications [5]. Zika was declared a Public Health Emergency of International Concern by the World Health Organization (WHO) in 2016 [6]. In Honduras, it was associated with an increase in microcephaly cases and a 10% case fatality rate for patients who developed Guillain–Barre Syndrome [7, 8]. In 2019, Honduras faced one of its worst Dengue outbreaks with > 18,000 cases until Epidemiological Week (EW) 26 and was declared a national emergency, disproportionately affecting pediatric patients. During COVID-19 pandemic, Dengue infection remained high, whereas, Chikungunya and Zika infections decreased. In 2022, the proportion of severe Dengue cases in Honduras was 1.72% higher than the American regional average of 0.26% [3, 9]. In Honduras, these arboviruses are transmitted by the same vectors, *Aedes aegypti* and *A. albopictus,* coexisting in the same geographical areas and presenting similar epidemiological behavior [3].

Arbovirus case notification is mandatory by the Honduran Ministry of Health (HMOH) and cases are reported weekly to the National Epidemiological Surveillance System (NESS) [10]. However, most reported cases are not confirmed by any laboratory method as physicians base their diagnoses on their clinical judgment and the HMOH guidelines [11]. It is estimated that < 1% of arboviral cases are confirmed by a laboratory method [3, 12, 13]. The laboratory confirmation of arboviruses is limited due to inadequate laboratory capacity and infrastructure, as well as a lack of proper transportation system for the routine shipment of blood samples. Moreover, when patients visit primary healthcare during the initial clinical stage, disease symptoms are often mild, non-specific, and not easily differentiated from other arboviral or non-arboviral febrile illness [14], contrary to arboviral patients in later clinical stages that display more distinguishable clinical features that facilitate diagnosis. These may contribute to physicians’ misdiagnosis of arboviral diseases leading to repercussions such as inadequate treatment and preventable complications.

Therefore, this study assessed the accuracy of physicians’ clinical diagnoses of Dengue, Chikungunya, and Zika in primary healthcare centers. We compared the diagnoses to their respective reverse transcription-quantitative PCR (RT-qPCR) results to determine the proportion of misdiagnoses. The epidemiological curves of clinical cases and RT-qPCR confirmed cases were graphed to observe if the clinical diagnoses reflected the actual behavior of the arboviral diseases.

## Methods

### Study setting

**Fig. 1:**
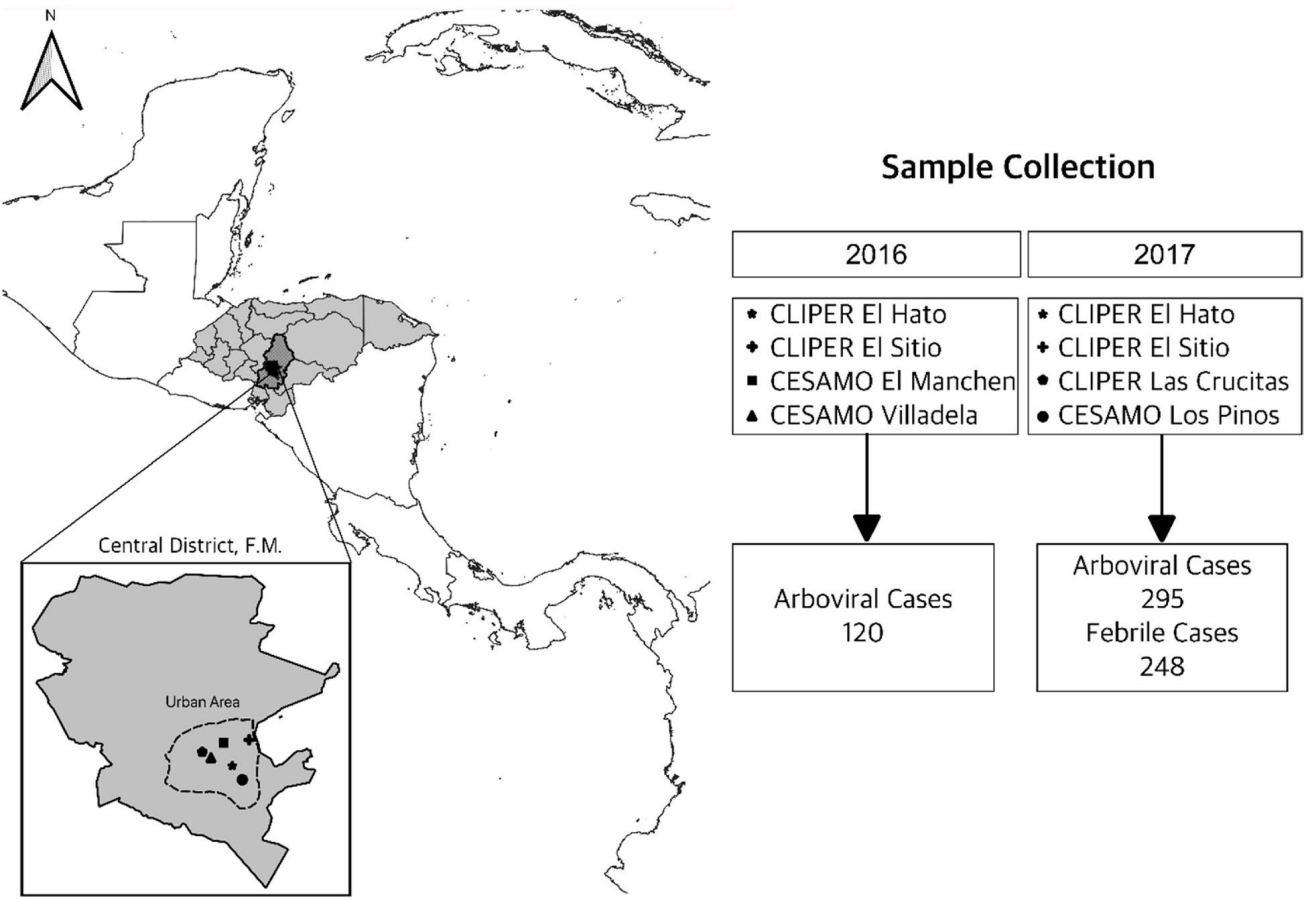
Geographic distribution of selected healthcare centers and sample collection. CLIPER: 24/7 Peripheric Clinic, CESAMO: Health Center with medical doctor. Map was generated using QGIS 3.10® software (https://download.qgis.org)

This was a cross-sectional, multi-center study using convenience sampling at urban primary healthcare centers in the Metropolitan Health Area (MHA) of the Central District during the epidemic season. The MHA (Tegucigalpa and Comayaguela) has the highest arboviral burden in Honduras [12, 13]. Arboviral infections are unevenly distributed in the Metropolitan Health Area due to geographical and infrastructural factors. In addition, there are challenges with accessibility to some health centers. To ensure sufficient sampling during the epidemic period, we used convenience sampling to select health centers with the highest reports of arboviral cases. El Hato, El Sitio, El Manchen, Villadela, Las Crucitas, and Los Pinos primary healthcare centers, which reported the highest arboviral case incidence in the MHA, were selected using NESS data. To illustrate the study setting, a GIS-based map was generated using QGIS® 3.10 software (Fig. 1).

### Case description

A Dengue, Chikungunya, or Zika clinical case was defined as a patient diagnosed with Dengue, Chikungunya, or Zika by a physician, respectively. Arbovirus clinical guidelines used by physicians, such as those for Dengue, exhibit high sensitivity (> 90%) but low specificity (< 50%) [15]. The Zika clinical guidelines are more specific than the other arboviruses, but exhibit similar sensitivity and specificity [16]. In contrast, a Dengue, Chikungunya, or Zika confirmed case was defined as a patient with RT-qPCR confirmation. Arboviral clinical cases and arboviral confirmed cases are the sum of clinical and confirmed cases, respectively. A febrile case is a patient diagnosed by a physician with non-arboviral viral illness.

### Patient enrollment and data collection

Consecutive and incident cases of Dengue, Chikungunya, or Zika diagnosed within five days of disease onset from all ages were recruited from August to October 2016 and July to September 2017. A sample size of 382 was calculated using Raosoft® sample size calculator, providing a 95% confidence level from the 54,310 arboviral cases in the reported Metropolitan Health Region in the year 2015. Clinical information (patient’s demographic, clinical, and laboratory data) and dried blood spots (DBSs) were collected by trained physicians. In total, 120 and 295 arboviral clinical cases were enrolled in 2016 and 2017, respectively. Furthermore, 248 febrile patients were obtained in 2017.

### Dried Blood Spots 

Physicians were trained to properly collect and store the DBSs. Whatman 903 filter paper was used to collect three drops of capillary finger-prick blood from adults and heel-pricked blood from small children. DBSs were completely air-dried and stored at room temperature in individual sealed plastic bags with a desiccant. Samples were properly packaged and shipped to the Department of Tropical Medicine at National Yang Ming Chiao Tung University (NYCU), Taiwan, R.O.C. for molecular analysis.

### RT-qPCR

A 6-mm disc (16 μl of blood) punched from the DBS was used for RNA extraction using a QIAGEN Viral RNA extraction kit (Qiagen®, Hilden Germany). The DENV, CHIKV, and ZIKV primers used were according to Shu et al. [17] Pastorino et al. [18], and Avila et al. [19], respectively. The SYBR green one-step RT-qPCR was performed under the same thermocycling conditions to detect all three arboviruses using a QIAGEN Quantinova One-Step RT-PCR kit (Qiagen®, Hilden Germany) following manufacturer’s instruction. All positive samples were checked by melting point and sequenced at the Genomic Research Center in NYCU. Full protocol is available at https://www.protocols.io/view/rna-isolation-and-RT-PCR-for-dengue-chikungunya-a-bcwyixfw.

### Statistical analysis

Binary and multinomial regressions comparing arboviral clinical and confirmed cases were fitted to estimate adjusted odds ratio (OR) with 95% confidence interval (CI). To avoid overfitting, forward and backward stepwise selection logistic regression were applied. The optimal model with the smallest Akaike’s Information Criterion value was chosen. Arboviral co-infections and incomplete blood counts were excluded. A *p*-value < 0.05 was considered statistically significant. All data analyses were conducted using STATA® version 16 and GraphPad Prism® 7.

## Results

### Demographic characteristics of the study population by clinical diagnosis

To evaluate the accuracy of physicians’ clinical diagnoses in Honduras, clinical information and DBSs of 415 arboviral cases and 248 febrile cases were collected from six urban primary healthcare centers in the MHA (Fig. 1). Healthcare center selection and patient enrollment flowcharts are shown in Figs. S1 and S2 respectively. Most arboviral cases were aged 21–40 years (38.31%), followed by 6–20 years (30.84%), as shown in Table 1. However, in febrile cases, 35.48% were children aged 0–5 years compared to 10.36% in the arboviral cases.

**Table 1:** Demographic characteristics of the study population based on physicians’ clinical diagnosis

	Dengue		Chikungunya		Zika		Total Arbovirus^a^		Febrile	
	*n* = 316		*n* = 51		*n* = 48		*n* = 415		*n* = 248	
	n	%	n	%	n	%	n	%	n	%
**Age**										
0–5 years	42	13.29	1	1.96	0	0	43	10.36	87	35.08
6–20 years	105	33.23	8	15.69	15	31.25	128	30.84	65	26.21
21–40 years	114	36.08	24	47.06	21	43.75	159	38.31	71	28.36
> 40 years	55	17.41	18	35.29	12	25.00	85	20.48	25	10.08
**Gender**										
Male	120	37.97	17	33.33	19	39.58	156	37.59	97	39.11
Female	196	62.03	34	66.67	29	60.42	259	62.41	151	60.89
**Area of Residence**										
Urban	279	88.29	45	88.24	38	79.17	362	87.23	223	89.92
Rural	37	11.71	6	11.76	10	20.83	53	12.77	25	10.08
**Education Level**										
Not apply^b^	46	14.56	4	7.84	4	8.33	54	13.01	87	35.08
School/College student	109	34.49	13	25.49	14	29.17	136	32.77	68	27.42
Incomplete Primary	30	9.49	5	9.8	3	6.25	38	9.16	18	7.26
Complete Primary	31	9.81	6	11.76	8	16.67	45	10.84	22	8.87
Incomplete High school	40	12.66	11	21.57	3	6.25	54	13.01	19	7.66
Complete High school or higher	60	18.99	12	23.53	16	33.33	88	21.2	34	13.71

^a^Total Arbovirus is the sum of all Dengue, Chikungunya, and Zika clinical cases from 2016 and 2017

^b^Patients younger than 6 years old that have not initiated school education

### Comparison between physicians’ clinical diagnoses and RT-qPCR confirmations

For accuracy assessment, a diagnosis was deemed correct if the physician’s arboviral clinical diagnosis matched the corresponding RT-qPCR result. Table S2 displays the accuracy measures for the three arboviral clinical diagnoses during both years. High disagreement between physicians’ clinical diagnoses and the RT-qPCR confirmations was observed (Table 2). In 415 arboviral clinical cases (2016–2017), only 30.84% were RT-qPCR positive. Of 316 Dengue clinical cases, only 26 (8.23%) were confirmed Dengue, while 59 (18.67%) were Zika, and 4 (0.91%) were Dengue and Zika co-infected. Interestingly, in 51 Chikungunya clinical cases, none were confirmed Chikungunya, but 12 (25.49%) and 3 (5.88%) cases were confirmed Zika and Dengue, respectively. In 48 Zika clinical cases, 13 (27.08%) were confirmed Zika, while 3 (6.25%) and 3 (6.25%) were Dengue and Chikungunya, respectively. Interestingly, of the 248 febrile cases in 2017, a high proportion (20.96%) were confirmed arboviral infections which 31 (12.5%) were Dengue, 16 (6.45%) were Zika, and 4 (1.61%) were Dengue and Zika co-infection. In 2017, children 0–5 years had more RT-qPCR positive results in the febrile cases (20, 38.46%) than in the arboviral clinical cases (11, 19.3%) (Table S1). We also compared the number of PCR-positive arboviral cases based on the days of symptom onset and determined that the percentage of arboviral-positive cases was similar, regardless of the day of onset. The chi-square for trend was not significant (Table S3). Day 3 had the highest number of PCR-positive cases but its proportion was similar to other days. In addition, no significant association was observed between the day of symptom onset nor the percentage of accurate diagnosis for any arbovirus (Table S4).

**Table 2:** Diagnostic comparison between physicians’ clinical diagnosis and SYBR RT-qPCR detection

**Clinical Diagnosis**		**RT-qPCR detection**									
	**Total**	**Dengue**		**Chikungunya**		**Zika**		**Dengue/Zika**		**Negative**	
	N	n	%	n	%	n	%	n	%	n	%
Total Arbovirus^a^	415	32	7.27	7	1.69	85	20.48	4	0.96	289	69.16
Dengue	316	26	8.23	4	1.27	59	18.67	4	1.27	223	70.56
Chikungunya	51	3	5.88	0	0.00	13	25.49	0	0.00	35	68.63
Zika	48	3	6.25	3	6.25	13	27.08	0	0.00	29	60.42
Febrile	248	31	12.5	1	0.40	16	6.45	4	1.61	196	79.03

^a^Total Arbovirus is the sum of all Dengue, Chikungunya, and Zika clinical diagnoses from 2016 and 2017

### Evaluation of physicians’ adherence with the HMOH case definitions

**Fig. 2:**
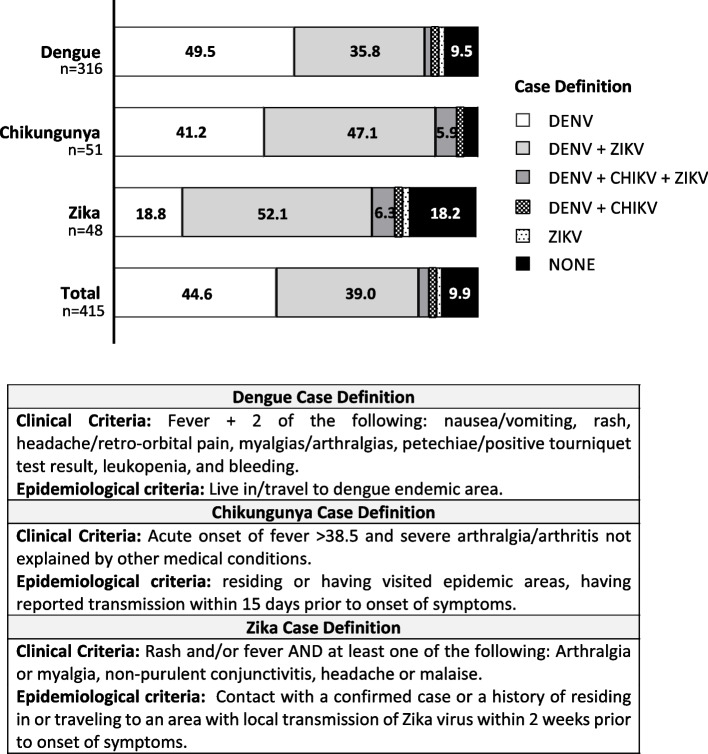
Evaluation of the physicians’ clinical diagnosis that fulfills the MOH case definition

Due to the high proportion of misclassified arboviral clinical cases, we reviewed the patients’ clinical manifestations to determine whether physicians followed the HMOH case definitions [11] (Fig. 2). Among the 316 Dengue clinical cases, 49.5% solely fulfilled the Dengue case definition, whereas 41% simultaneously fit Dengue and other arboviral case definitions. Interestingly, for the 51 Chikungunya clinical cases, none fulfilled solely the Chikungunya case definition, but 41.2% fulfilled the Dengue case definition alone, while 53% fit not only Dengue but also other arboviral case definitions. Only 2.08% of the 48 Zika clinical cases exclusively fit the Zika case definition, while 58.4% simultaneously fulfilled Zika with other arboviral case definitions.

### Relevant clinical manifestations to differentiate arboviral and febrile cases

Logistic regression, using the febrile cases as reference, was utilized to investigate clinical manifestations for better distinguishing arboviral infections. Out of 663 patients, 197 were excluded due to incomplete blood counts, and 8 were excluded due to Dengue/Zika co-infections. As shown in Table 3A, conjunctivitis (OR 4.09, 95% CI 1.75–9.52), retro-orbital pain (OR 3.34, 95% CI 1.76–6.31), arthralgia (OR 3.25, 95% CI 1.64–6.46), and myalgia (OR 2.03, 95% CI 1.02–4.07) were positively correlated with physicians’ arboviral diagnoses and matched the HMOH case definitions. In contrast, cough, pharyngeal hyperemia, and abdominal pain were negatively associated. Individually, for Dengue cases, conjunctivitis (OR 3.19, 95% CI 1.31–7.81), retro-orbital pain (OR 3.04, 95% CI 1.62–5.73), and arthralgia (OR 2.84, 95% CI 1.46–5.52) were the most significant. For Chikungunya cases, arthralgia (OR 27.44, 95% CI 5.58–134.89), followed by photophobia (OR 5.73, 95% CI 1.65–19.93), and dizziness (OR 3.94, 95% CI 1.38–11.28) were the most significant. Finally, rash (OR 25.43, 95% CI 6.18–104.55) and conjunctivitis (OR 12.78, 95% CI 3.24–50.35) were significant for Zika cases (Table 3A). Many of these clinical manifestations matched those present in their respective HMOH case definitions.

**Table 3:** Most relevant clinical features by clinical diagnosis and RT-qPCR confirmation, for differentiating arboviral infections from other febrile infections

**A. Clinical Diagnosis**												
**Binary**^**a**^				**Multinomial**								
**Arbovirus**				**Dengue**			**Chikungunya**			**Zika**		
**Variables**		**OR**	**CI 95%**	**Variables**	**OR**	**CI 95%**	**Variables**	**OR**	**CI 95%**	**Variables**	**OR**	**CI 95%**
Arthritis		7.49	0.59–95.97	Conjunctivitis^d^	3.19^*^	1.31–7.81	Arthralgia^b,c,d^	27.44^*^	5.58–134.89	Rash^d^	25.43^*^	6.18–104.55
Conjunctivitis^d^		4.09^*^	1.76–9.52	Retro orbital pain^b^	3.04^*^	1.62–5.73	Photophobia	5.73^*^	1.65–19.93	Conjunctivitis^d^	12.78^*^	3.24–50.35
Retro orbital pain^b^		3.34^*^	1.76–6.31	Arthralgia^b,c,d^	2.84^*^	1.46–5.52	Dizziness	3.94^*^	1.38–11.28	Arthralgia^b,c,d^	2.97	0.89–9.88
Arthralgia^b,c,d^		3.25^*^	1.64–6.46	Rash^d^	2.48	0.80–7.65	Rash^d^	3.31	0.86–12.77	Photophobia	2.83	0.67–11.88
Rash^d^		2.62	0.89–7.71	Photophobia	2.07	0.69–6.19	Conjunctivitis	2.73	0.90–8.27	Retro orbital pain^b^	1.25	0.41–3.87
Myalgia		2.03^*^	1.02–4.07	Dizziness	1.01	0.51–2.02	Retro orbital pain^b^	2.42	0.96–6.13	Hematocrit	1.24^*^	1.09–1.43
Platelets		0.99^*^	0.98–0.99	Age	1.01	0.99–1.02	Age	1.03^*^	1.00–1.06	Dizziness	1.19	0.37–3.85
White Blood Cells		0.77^*^	0.69–0.86	Hematocrit	1.01	0.94–1.08	Neutrophils	1.02^*^	1.00–1.04	Neutrophils	1.05^*^	1.02–1.08
Anorexia		0.55	0.25–1.21	Neutrophils	1.01	0.99–1.02	Hematocrit	0.99	0.89–1.10	Age	1.03^*^	1.00–1.06
Abdominal pain		0.40^*^	0.21–0.76	Platelets	0.98^*^	0.97–0.99	Platelets	0.99	0.99–1.00	Platelets	0.99	0.98–1.00
Cough		0.26^*^	0.07–0.96	White Blood Cells	0.78^*^	0.71–0.86	White Blood Cells	0.79^*^	0.69–0.91	White Blood Cells	0.75^*^	0.62–0.91
Pharyngeal Hyperemia		0.02^*^	0.01–0.21	Abdominal pain	0.47^*^	0.25–0.89	Abdominal pain	0.29^*^	0.11–0.72	Abdominal pain	0.44	0.14–1.35
				Pharyngeal Hyperemia	0.03^*^	0.01–0.28	Pharyngeal Hyperemia	NA		Pharyngeal Hyperemia	NA	
**B. RT-qPCR confirmation**												
**Arbovirus**				**Dengue**			**Chikungunya**			**Zika**		
**Variables**	**OR**		**CI 95%**	**Variables**	**OR**	**CI 95%**	**Variables**	**OR**	**CI 95%**	**Variables**	**OR**	**CI 95%**
Pharyngeal Hyperemia	4.76^*^		1.49–15.25	Hematocrit	1.16	0.98–1.37	Photophobia	2.33	0.50–10.89	Photophobia	2.26^*^	1.29–3.97
Cough	3.70^*^		1.07–12.84	White Blood Cells	1.08	0.98–1.37	Hematocrit	1.40	0.85–2.30	Hematocrit	1.40^*^	1.18–1.66
Photophobia	1.72^*^		1.06–2.79	Platelets	0.99^*^	0.99–0.99	Platelets	1.00	0.99–1.01	White Blood Cells	0.99	0.91–1.09
Hematocrit	1.28^*^		1.12–1.45	Lymphocytes	0.99	0.99–1.00	Lymphocytes	0.96	0.92–1.01	Platelets	0.99^*^	0.99–0.99
Platelets	0.99^*^		0.99–0.99	Photophobia	0.78	0.36–1.67	White Blood Cells	0.61	0.37–1.02	Lymphocytes	0.98^*^	0.96–0.99
Lymph nodes	0.55		0.29–1.04	Hemoglobin	0.68	0.42–1.11	Hemoglobin	0.45	0.11–1.87	Vomit	0.54^*^	0.31–0.97
Bloating	0.52		0.26–1.07	Vomit	0.50	0.25–1.01	Vomit	0.38	0.07–2.16	Hemoglobin	0.39^*^	0.24–0.63
Hemoglobin	0.51^*^		0.35–0.74									
Vomit	0.48^*^		0.30–0.78									

^*^P-value < 0.05

^^^*NA* Not available. The odds ratio of these variables cannot be calculated due to the small number of events.

^a^Backward Stepwise Binary Logistic Regression, reference group febrile cases

^b^Dengue Criteria

^c^Chikungunya Criteria

^d^Zika Criteria

On the other hand, the same analysis using the RT-qPCR confirmed cases showed that the relevant clinical manifestations differ from the clinical cases (Table 3B). For arboviral confirmed cases, photophobia (1.72 1.06–2.79) and higher hematocrit (1.28 1.12–1.45) were correlated. Pharyngeal hyperemia (4.76, 95% CI 1.49–15.25) and cough (3.70 95% CI 1.07–12.84) were positively associated but negatively associated in the clinical cases. Higher hemoglobin, lymphadenitis, and vomit were negatively associated.

None of Dengue hallmarks like conjunctivitis, retro-orbital pain, and arthralgia, were significant in the Dengue confirmed cases. Higher hematocrit (OR 1.16, 95% CI 0.98–1.37) and higher white blood cells (OR 1.08, 95% CI 0.98–1.37) were positively correlated. No significant clinical manifestations were observed for Chikungunya due to only eight confirmed cases. In Zika confirmed cases, photophobia (OR 2.26, 95% CI 1.28–3.97) and higher hematocrit (OR 1.40, 95% CI 1.17–1.65) were significant.

### Complementary criteria used for arboviral clinical diagnosis

Besides case definitions and clinical manifestations, other criteria used by physicians for diagnosis was recorded. As shown in Table 4, from 363 arboviral clinical cases with complete clinical data, 30.58% of physicians used only symptom compliance (Patients whose clinical symptoms comply with an arboviral infection) for diagnosis, whereas, 40.77% used symptom compliance plus patients with suspected close contact (PSCC). Less utilized criteria included patients residing in area with local outbreak (7.99%) and compatible hemogram results (10.47%).

**Table 4:** Complemental criteria utilized by physicians to diagnose arboviral infections

Complemental Criteria	Arbovirus^a^		Dengue^a^		Chikungunya^a^		Zika^a^	
	n	%	n	%	n	%	n	%
	363	100	280	100	48	100	35	100
SC	111	30.58	84	30.00	14	29.17	13	37.14
SC + PSCC	148	40.77	105	37.50	23	47.92	20	57.14
SC + HC	38	10.47	35	12.50	2	4.17	1	2.86
SC + LO	29	7.99	25	8.93	3	6.25	1	2.86
SC + PSCC + HC	14	3.86	11	3.93	3	6.25	0	0.00
SC + PSCC + LO	10	2.75	7	2.50	3	6.25	0	0.00
SC + HC + LO	11	3.03	11	3.93	0	0.00	0	0.00
SC + PSCC + HC + LO	2	0.55	2	0.71	0	0.00	0	0.00

Symptom compliance (SC): Patient whose clinical symptoms comply with an arboviral infection. Patient with suspected close contact (PSCC): Patient who reported close contact with a clinically diagnosed arboviral case in the last 2 weeks. Hemogram Compatible (HC): Patient presents hemogram findings compatible with an arboviral infection such as leukopenia, thrombocytopenia, among others. Local Outbreak (LO): Patient resides or frequently attends a specific area or neighborhood that has recently reported a high number of arboviral cases

^a^Cases with complete clinical data

### Temporal distribution of arboviral clinical and confirmed cases

**Fig. 3:**
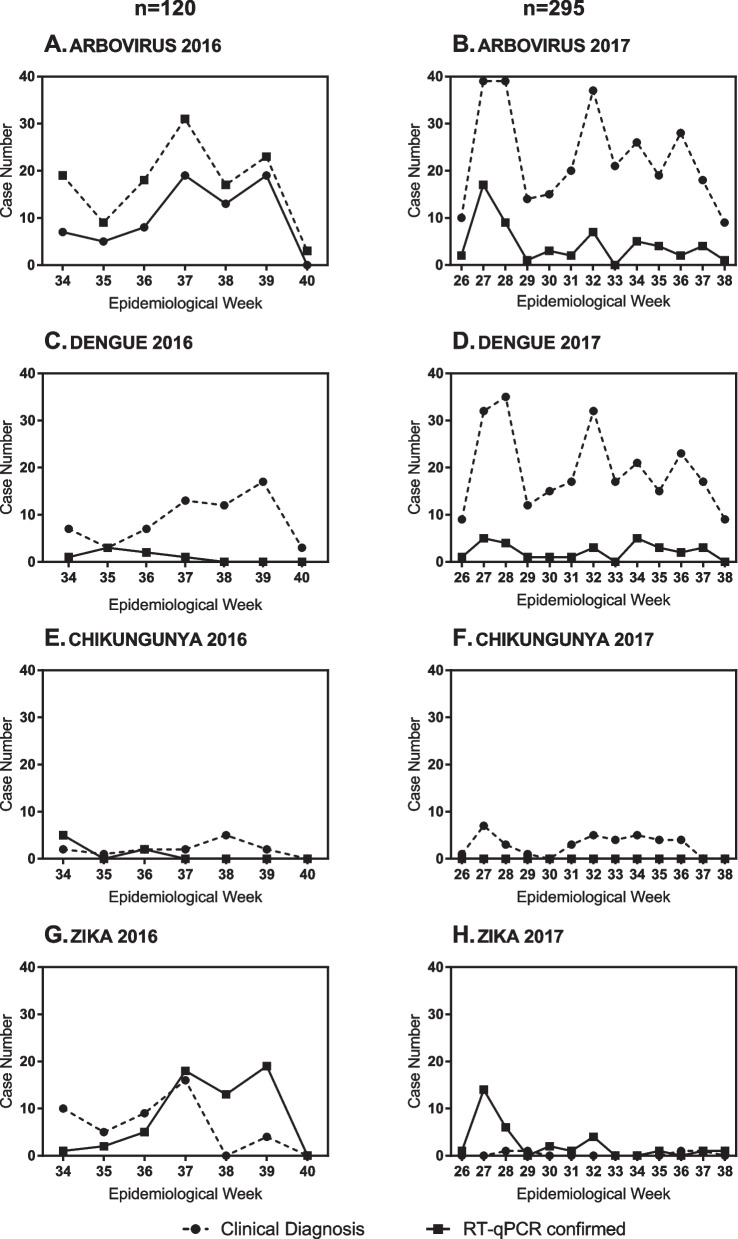
Temporal distribution of clinically diagnosed arboviral and RT-qPCR positive cases

We analyzed whether the clinical diagnoses reflect the actual epidemiological behavior of the arboviruses (Fig. 3). Clinical and confirmed cases from 2016 and 2017 (excluding the febrile cases) were distributed by epidemiological week (EW) to graph their epidemiological curve (EC). The EC of arboviral confirmed cases was roughly parallel to the arboviral clinical cases in both years. There were two prominent peaks on EW 37 and 39 in 2016 (Fig. 3A), whereas four peaks in 2017 (Fig. 3B).

However, discrepancies were observed when comparing the ECs of Dengue, Chikungunya, and Zika clinical cases with their respective ECs of confirmed cases. Surprisingly, in 2016 no EC for any of the arboviral confirmed cases matched the corresponding EC of its clinical cases. Although Dengue clinical cases showed an increase between EW 37 to 39, the pattern of the confirmed cases did not match (Fig. 3C). Interestingly, the Zika clinical cases showed one prominent peak on EW 37 then diminished, but the confirmed Zika cases’ EC showed a high case number after EW 37 (Fig. 3G). Chikungunya clinical cases were overestimated (Fig. 3E).

In 2017, the EC of the Dengue confirmed cases was somewhat parallel to the clinical case group (Fig. 3D), but the number of confirmed cases was lower than expected. Again, Chikungunya clinical cases were overestimated, with 0 confirmed cases (Fig. 3F). Almost no Zika clinical cases were reported, but two peaks of confirmed cases were observed in EW 27 and 32 (Fig. 3H).

## Discussion

This study highlights the low diagnostic accuracy of primary care physicians for Dengue, Chikungunya, and Zika in the MHA of Honduras. Patients with similar clinical symptoms may have had viral infections caused by non-arboviral pathogens, which could have affected the differential diagnosis and contributed to misdiagnosis. Children aged < 0–5 years had more arboviral confirmed cases from the febrile cases than the arboviral clinical cases. This could be explained because WHO and PAHO case definitions often miss pediatric arboviral cases since their manifestations are mild, non-specific, and vary with age [15, 20]. Furthermore, small children cannot clearly describe their symptoms, and other viral illnesses are common at this age, which might dissuade physicians from diagnosing arboviral infections [15]. Delayed diagnosis and treatment of arboviruses in small children can result in increased complications and mortality [21].

The case definitions of these arboviruses frequently overlap in co-endemic areas, as more than 40% of the arboviral clinical cases in our study fitted more than one case definition. A study in Nicaragua, a country neighboring Honduras, showed that about 75% of RT-qPCR confirmed Zika patients that fitted the WHO Zika case definition also fitted the Dengue definition [20]. Furthermore, the WHO Dengue case definitions based on hospitalized patients’ data [22] may not apply to patients attending primary healthcare. Godaert et al. demonstrated that atypical clinical presentations of Chikungunya, like no fever or joint pain, in older adults are frequent and 42.7% could not be classified by WHO case definition [23]. Other limitations of using case definitions include lack of uniform application, varying sensitivity depending on the institution’s criteria, and lack of generalization towards specific populations, like small children and older adults [15, 20, 24]. Therefore, physicians might misclassify these arboviruses when solely using case definitions [15].

According to our regression models, most of the significant clinical manifestations of arboviral confirmed cases were not the ones present in their corresponding HMOH case definitions. Lower platelets, lower leukocytes, and lower lymphocyte counts were significant in arboviral confirmed cases as reported in previous studies [25, 26], but did not reach the values for thrombocytopenia, leukopenia, and lymphopenia, respectively. These could be explained because patients often attend primary care during early stages of infection when the typical arboviral clinical manifestations are not yet present, whereas patients with more severe clinical symptoms or warning signs might directly attend hospitals.

Clinical manifestations like cough and pharyngeal hyperemia were found positively correlated in the arboviral confirmed cases, but negatively correlated in the arboviral clinical cases. Upper tract respiratory manifestations might confound physicians in diagnosing arboviral infections as respiratory illnesses.

Most studies on the diagnostic accuracy of arboviruses focus on the case definitions [15, 25–27], our study focused on the physicians’ diagnosis. Besides symptom compliance, a patient with suspected close contact (PSCC) was observed in 44.63% of the arboviral clinical cases, indicating that epidemiological information influence diagnosis. PSCCs whose close contact was not laboratory-confirmed can lead to misdiagnosis, as all derived diagnoses could be erroneous, leading to systematic errors affecting arboviral surveillance data (Fig. 3). The accuracy of clinical diagnosis for arboviral infections is likely to be influenced by disease prevalence. In non-epidemic periods with low disease prevalence, the positive predictive value of clinical diagnosis may be reduced. Furthermore, physicians may be less likely to consider arboviral infections due to lack of PSCC and outbreak reports. Conversely, in high-prevalence settings, the pre-test probability of disease is increased, resulting in improved diagnostic accuracy and lower rates of missed diagnoses. Honduran physicians frequently requested hemograms when suspecting arboviral infections, just like physicians in Singapore [28]. However, less than 10% of Honduran physicians diagnosed arboviral infections based on the hemogram result as most results are inespecific at the time of the medical consultation.

The EC patterns of arboviral clinical cases and their corresponding confirmed cases were similar in both years, but not when analyzing Dengue, Chikungunya, and Zika separately. According to the confirmed cases ECs in both years, many Chikungunya and Zika cases were misclassified as Dengue. This phenomenon was also observed in a study in Roatan, Honduras where from all the clinically suspected Dengue cases collected, the EC of the RT-qPCR confirmed cases showed most peaks were Zika followed by Chikungunya [7]. In both years, physicians probably diagnosed more Dengue because they are more acquainted with this infection as it has been endemic in Honduras for longer time. When Zika was declared a public health emergency in 2016 [2], more Zika cases were diagnosed as physicians were more aware of it. Nevertheless, once the Zika emergency ended in 2017, the situation reverted to diagnosing Dengue. Physicians may be affected by the perceived epidemic situation at the time, explaining why Chikungunya kept being diagnosed despite almost no confirmed cases found.

These suggest that without routinely laboratory confirmation, the prevalence of arboviral infections based only on physician’s diagnosis can lead to reporting errors in the surveillance database. As Bautista et al. demonstrated, current Zika surveillance systems in Latin America had limited capacity to detect outbreaks without serological surveillance [29]. Periodic testing and feedback of these results to physicians must be done to improve diagnosis and surveillance data.

DBS approach is a cost-efficient alternative to facilitate laboratory confirmation [30]. To improve our detection method’s sensitivity, we collected capillary blood which has longer-lasting viremia than venous blood, selected patients on the first five days of infection, adequate sample storage to avoid RNA degradation, and utilized SYBR-Green One-step RT-qPCR [30]. Positive samples were double confirmed with sequencing to reduce false positives. The sensitivity for the RT-qPCR for Dengue, Chikungunya, and Zika on Whatman 903 filter paper was 16, 160, and 160 PFU/ml, respectively [19]. Furthermore, no correlation was found between the percentage of PCR-positive arboviral cases and the onset days of the disease. Nevertheless, our method’s sensitivity still may have failed to detect low viremia levels in patients with five or more days of infection, despite its high sensitivity. In 2016 we obtained a prevalence of 5.8%, 5.8%, and 48.3% for Dengue, Chikungunya, and Zika, respectively (Table S2). In the same year, a study in Roatan, Honduras, found a prevalence of 3% for Dengue, 5.83% for Chikungunya, and 43% for Zika [7]. Our results utilizing DBSs utilizing were comparable to theirs utilizing whole blood. This DBS method is sensitive enough to be used for surveillance. Moreover, substituting PCR with IgM and antigen testing may result in varying diagnostic accuracy rates, given their limited sensitivity. These tests are most effective during a specific timeframe following infection and can produce false-negative outcomes outside of this period. False-positive results may arise from cross-reactivity between Dengue and Zika, as well as between IgG and IgM antibodies [31]. While PCR may be considered the gold standard for arboviral diagnosis, it is often challenging to utilize this technique in rural and suburban regions of tropical areas. Therefore, it is crucial to improve the diagnostic criteria for arboviral infections for primary care with data of the local population. Filter paper can be an alternative for sample transportation. Additionally, establishing properly arranged PCR sentinel stations in highly endemic areas could help monitor the spread of arboviral epidemics and provide valuable information for disease control measures.

Since we used a short period for data and sample collection, convenience sampling, and one region, the present study cannot reflect physicians’ diagnostic accuracy across the whole country. Nevertheless, this study was multi-center and had a large sample size. Despite these limitations, the study still provides a snapshot of the magnitude of misdiagnosis and how it affects surveillance data. This problem is not particular to Honduras but to other low-income countries that rely on clinical diagnosis for surveillance.

## Conclusions

This study highlights the low accuracy of primary care physicians’ clinical diagnoses in the MHA of Honduras. Unspecific clinical manifestations, particularly in young children, overlapping case definitions, reported close contact with a suspected arboviral case, and vague hemogram results may contribute to misdiagnosis. Consequently, without laboratory confirmation, arboviral surveillance data may not reflect the actual epidemiological situation impacting national health policies. Case definitions and other alternative diagnostic tools like predictive models or scoring systems must be developed to improve clinical diagnosis for patients attending primary healthcare.

## Supplementary Information

**Additional file 1: Figure S1.** Healthcare center selection flowchart. **Figure S2.** Patient recruitment flowchart. **Table S1.** Age Distribution of RT-qPCR positive cases in 2017 from arboviral and febrile cases. **Table S2.** Accuracy measures for the clinical diagnosis of Dengue, Chikungunya, and Zika in 2016 and 2017. **Table S3.** Percentage of arboviral positive PCR cases by number of days from disease onset. **Table S4.** Number of accurate arboviral diagnoses by days of symptom onset.

## Data Availability

The datasets generated during and/or analyzed during the current study are not publicly available due to them containing information that could compromise research participant privacy/consent, but are available from the corresponding author on reasonable request.

## Abbreviations

EW: Epidemiological Week
EC: Epidemiological Curve
RT-qPCR: Reverse Transcription-Quantitative PCR
HMOH: Honduran Ministry of Health
NESS: National Epidemiological Surveillance System
MHA: Metropolitan Health Area
DBSs: Dried Blood Spots
NYCU: National Yang Ming Chiao Tung University
