# Supplementary material for: Accuracy of Dengue, Chikungunya, and Zika diagnoses by primary healthcare physicians in Tegucigalpa, Honduras
Source: BMC Infect Dis. 2023 Jun 1;23:371. doi: 10.1186/s12879-023-08346-1 (PMC10233517; doi:10.1186/s12879-023-08346-1)
Supplement: Supplementary file 1 — Additional file 1: Figure S1. Healthcare center selection flowchart. Figure S2. Patient recruitment flowchart. Table S1. Age Distribution of RT-qPCR positive cases in 2017 from arboviral and febrile cases. Table S2. Accuracy measures for the clinical diagnosis of Dengue, Chikungunya, and Zika in 2016 and 2017. Table S3. Percentage of arboviral positive PCR cases by number of days from disease onset. Table S4. Number of accurate arboviral diagnoses by days of symptom onset. [file 12879_2023_8346_MOESM1_ESM.pdf]

Figure S1. Healthcare center selection flowchart.

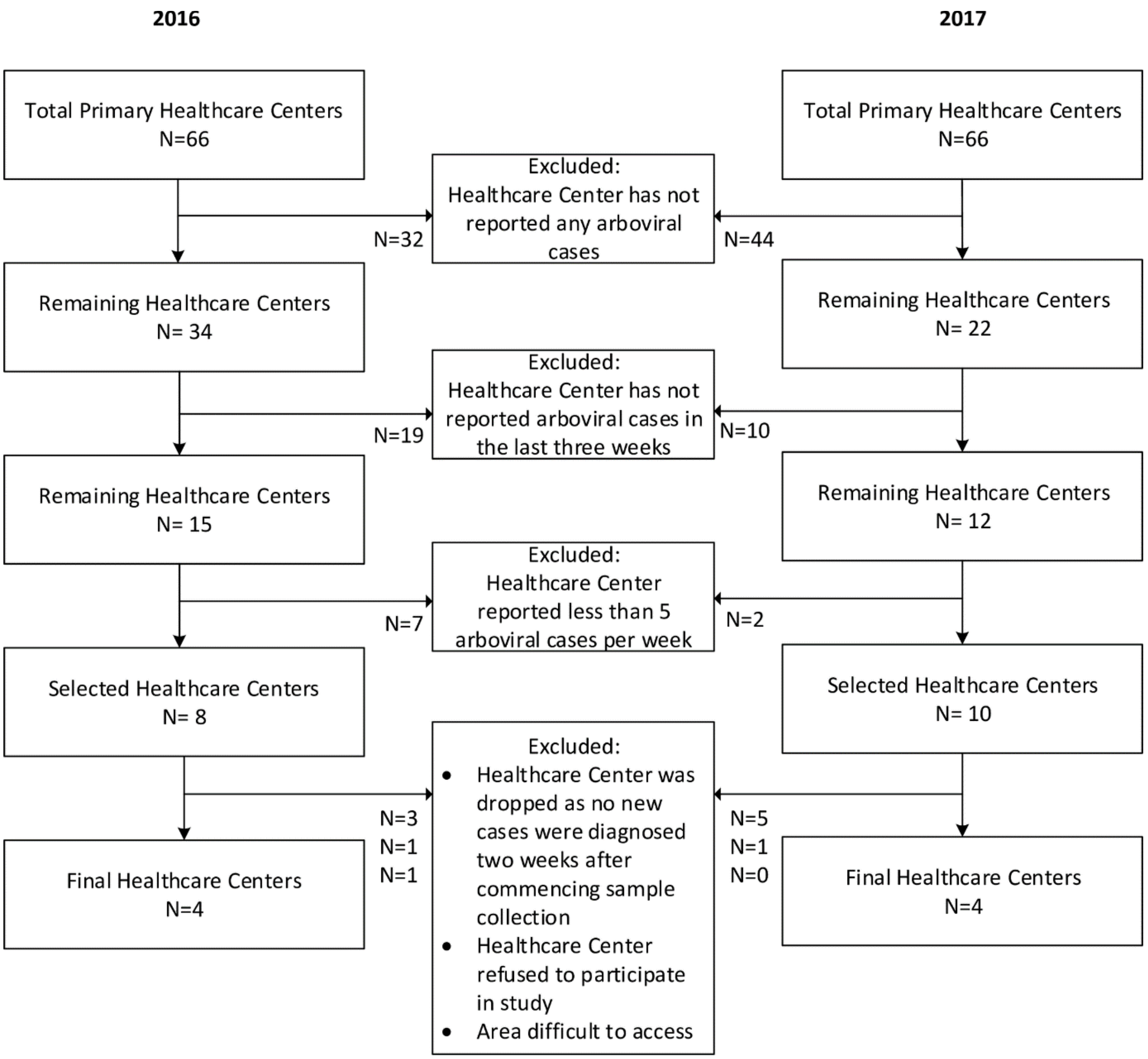

Figure S2. Patient recruitment flowchart.

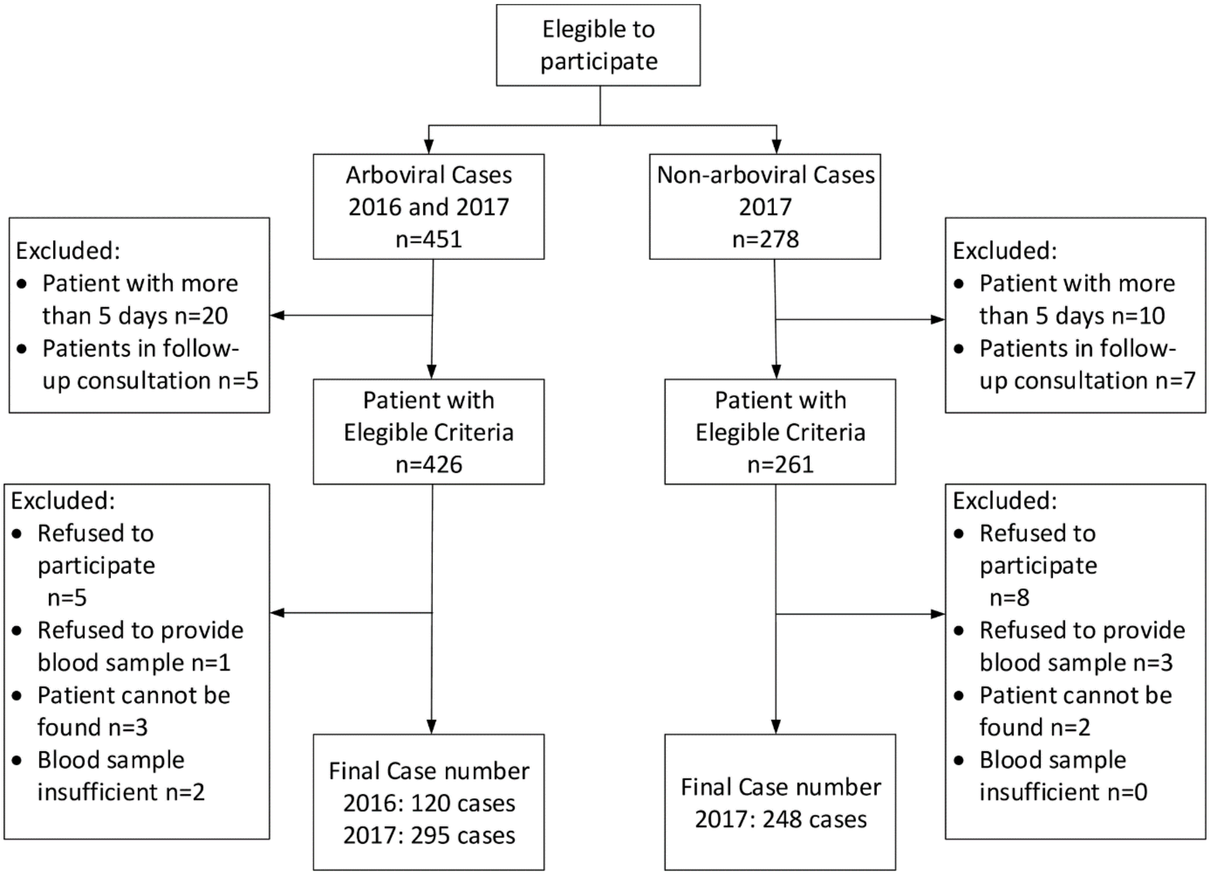

Table S1. Age Distribution of RT-qPCR positive cases in 2017 from arboviral and febrile cases

	Arboviral Cases n=295				Febrile Cases n=248			
	RT-qPCR +		RT-qPCR -		RT-qPCR +		RT-qPCR -	
**Age Group**	**n**	**%**	**n**	**%**	**n**	**%**	**n**	**%**
0-5 years	11	19.30	32	13.45	20	38.46	67	39.18
6-20 years	19	33.33	58	24.37	11	21.15	54	27.55
21-40 years	17	28.82	99	41.60	14	26.92	57	29.08
> 40 years	10	17.54	49	20.59	7	13.46	18	9.18
Total	57	100	238	100	52	100	196	100

Table S2. Accuracy measures for the clinical diagnosis of Dengue, Chikungunya, and Zika in 2016 and 2017

					Prevalence		Sensitivity		Specificity		PPV		NPV	
**Diagnosis**	**TP**	**FP**	**FN**	**TN**	**%**	**CI 95%**	**%**	**CI 95%**	**%**	**CI 95%**	**%**	**CI 95%**	**%**	**CI 95%**
**2016 n=120**														
DENV	3	59	4	54	5.8	2.4-11.6	42.9	9.9-81.6	47.8	38.3-57.4	4.8	1.0-14.0	93.1	83.3-98.0
CHIKV	0	14	7	99	5.8	2.4-11.6	0.0	0.0-41.0	87.6	80.1-93.1	0.0	0.0-23.0	93.4	86.9-97.0
ZIKV	13	31	45	31	48.3	39.1-57.6	22.4	12.5-35.6	50	37.0-63.0	29.5	16.8-45.0	40.8	29.6-53.0
**2017 n=295**														
DENV	27	227	2	39	9.8	6.7-13.8	93.1	77.2-99.2	14.7	10.6-19.5	10.6	7.1-15.0	95.1	83.5-99.0
CHIKV	0	37	258	0	NA		NA		NA		NA		NA	
ZIKV	0	4	31	260	10.5	7.3-14.6	0	0.0-11.2	98.5	96.2-99.6	0	0.0-60.0	89.3	85.2-93.0

TP: True Positive FP: False Positive FN: False Negative TN: True Negative PPV: Positive Predictive Value NPV: Negative Predictive Values CI: Confidence Interval

NA: Not Available

Table S3. Percentage of arboviral positive PCR cases by number of days from disease onset

	Arboviral Clinical Cases					Febrile Cases					Overall				
Symptom Onset	Total	RT-qPCR+	%	RT-qPCR-	%	Total	RT-qPCR+	%	RT-qPCR-	%	Total	RT-qPCR+	%	RT-qPCR-	%
0 day	4	1	25.00	3	75.00	1	1	100.00	0	0.00	5	2	40.00	3	60.00
1 day	58	21	36.21	37	63.79	91	21	23.08	70	76.92	149	42	28.19	107	71.81
2 days	97	25	25.77	72	74.23	66	15	22.73	51	77.27	163	40	24.54	123	75.46
3 days	123	37	30.08	86	69.92	50	4	8.00	46	92.00	173	41	23.70	132	76.30
4 days	69	25	36.23	44	63.77	20	5	25.00	15	75.00	89	30	33.71	59	66.29
5 days	64	19	29.69	45	70.31	20	6	30.00	14	70.00	84	25	29.76	59	70.24
Total	415	128	30.84	287	69.16	248	52	20.97	196	79.03	663	180	27.15	483	72.85
x^2 for trend			0.893					0.7147					0.5321		

PCR is positive for Dengue, Chikungunya or Zika.

Table S4. Number of accurate arboviral diagnoses by days of symptom onset

	Number of Accurate Diagnoses								Not Accurate		Total	
	DENV		CHIKV		ZIKV		Accurate					
Symptom Onset	n	%	n	%	n	%	n	%	n	%	n	%
0	0	0.0	0	0.0	0	0.0	0	0.0	5	100.0	5	100.0
1	7	5.0	0	0.0	3	2.2	10	7.2	139	93.3	149	100.5
2	8	5.3	0	0.0	4	2.6	12	7.9	151	92.6	163	100.6
3	9	5.6	0	0.0	2	1.2	11	6.8	162	93.6	173	100.4
4	2	2.3	0	0.0	1	1.2	3	3.5	86	96.6	89	100.1
5	4	5.2	0	0.0	3	3.9	7	9.1	77	91.7	84	100.8
x^2 for trend	0.7676		--		0.7839		0.9243					

Accurate diagnosis means both clinical diagnosis and RT-qPCR match for the same arboviral disease.
